# Differentiation of mesenchymal stem cells into cardiomyocytes is regulated by miRNA-1-2 via WNT signaling pathway

**DOI:** 10.1186/s12929-017-0337-9

**Published:** 2017-05-10

**Authors:** Xing Shen, Bo Pan, Huiming Zhou, Lingjuan Liu, Tiewei Lv, Jing Zhu, Xupei Huang, Jie Tian

**Affiliations:** 1 Department of Cardiology, Heart Centre, The Children’s Hospital of Chongqing Medical University, 136 Zhongshan Er Road, Chongqing, 400014 Yu Zhong District China; 2 Department of Pediatrics, the Affiliated Hospital of Southwest Medical University, LuZhou, Sichuan 646000 China; 3 Department of Biomedical Science, Charlie E. Schmidt College of Medicine, Florida Atlantic University, Boca Raton, FL 33431 USA

**Keywords:** miR1-2, Mesenchymal stem cells, Cardiomyocyte differentiation, Wnt/β-catenin signaling pathway

## Abstract

**Background:**

Bone marrow derived stem cells (BMSCs) have the potential to differentiate into cardiomyocytes, but the rate of differentiation is low and the mechanism of differentiation is unclear completely. Here, we aimed to investigate the role of miR1-2 in differentiation of mouse BMSCs into cardiomyocyte-like cells and reveal the involved signaling pathways in the procedure.

**Methods:**

Mouse BMSCs were treated with miR1-2 and 5-azacytine (5-aza). The expression of cardiac cell markers: NKx2.5, cTnI and GATA4 in BMSCs were examined by qPCR. The apoptosis rate was detected by flow cytometry and the activity of the Wnt/β-catenin signaling pathway was evaluated by measuring the upstream protein of this signaling pathway.

**Results:**

After over-expression of miR1-2 in mouse BMSCs, the apoptosis rate was significantly lower than the 5-aza group, while the expressions of cardiac-specific genes: such as Nkx2.5, cTnI and GATA4 were significantly increased compared to the control group and the 5-aza group. Meanwhile, over-expression of miR1-2 in mouse BMSCs enhanced the expression of wnt11, JNK, β-catenin and TCF in the Wnt/β-catenin signaling pathway. Use of LGK-974, an inhibitor of Wnt/β-catenin signaling pathway, significantly reduced the expression of cardiac-specific genes and partially blocked the role of the miR1-2.

**Conclusion:**

Over-expression of miR1-2 in mouse BMSCs can induce them toward promoted cardiomyocyte differentiation via the activation of the Wnt/β-catenin signaling pathway. Compared to 5-aza, miR1-2 can induce differentiation of BMSCs into cardiomyocytes more effectively with a less cytotoxicity.

## Background

Cardiovascular diseases are the number one among the disease-caused mortality in the world. The use of stem cells as a potential therapeutic modality has recently been proposed for the treatment of cardiovascular diseases [1, 2]. Embryonic stem cells (ESCs) have been reported to be able to repair the damaged heart muscle and exhibit strong therapeutic potentials [1]. However, the ethical controversies on the origin of ESCs hinder its broad application in human patients. Emerging evidence has shown that bone marrow-derived mesenchymal stem cells (BMSCs) possess lots of important biological properties such as trans-differentiation, angiogenesis, anti-apoptosis, anti-fibrosis, anti-inflammatory, and immunosuppressing [2, 3]. In fact, BMSCs can turn into a variety of cell types such as cardiomyocytes, osteoblasts, endothelial cells, neurons, and fat cells due to their multi-potent differentiation abilities [2, 3]. But despite the growing attention paid to BMSCs in cardiac repairing,there are still many problems, one of them is the poor differentiation rate of BMSCs into myocardial cells. It has been reported that BMSCs were able to transdifferentiate into cardiomyocyte-like cells after being exposed to 5-azacytine (5-aza) [4], however, 5-aza is an exogenous chemical reagent that would cause cell damage. Therefore, it is very important to find a safe method for inducing differentiation of BMSCs into cardiomyocyte.

Micro ribonucleic acids (microRNAs, miRs) are endogenous short non-coding RNAs. They have important regulatory functions in a wide range of biological processes, including regulating cell proliferation, apoptosis, and aging [5]. In addition, they are also involved in the development of the heart and the regeneration of the myocardium [6, 7]. Recently, emerging evidence showed that miRNAs were closely associated with stem cells differentiation. It was reported that in cardiac progenitor cells, the over-expression of miR1 and miR499 reduced the rate of cell proliferation and enhanced the differentiation via repressing the HDAC4 or Sox6 [8]. Both miR1 and miR133 levels were increased in the differentiated ESCs, and miR1 was able to promote ESCs differentiation into cardiac lineage, however, miR133 may block the differentiation of myogenic precursors [9]. In mammals, miR1 has two copies, one is for miR1-1, mainly related to skeletal muscle development [10], one is for miR1-2, mainly involved in myocardial development [11]. Therefore, we speculate that miR1-2 plays a major role in differentiation of BMSCs into cardiomyocyte, but few studies have been done on it.

The miR1 has been reported to be able to modulate cardiomyogenesis and maintain the expression of muscle genes via down regulating the Notch or STAT3 signaling pathways [12–14]. While, Huang F et al found there was only downregulation of Hes-1,the downstream target molecule of the Notch pathway, but not regulation upstream molecules of the Notch pathway [14]. Because Notch signaling is initiated via ligand-Notch receptor interactions on neighboring cells [15]. It suggested other signaling pathway may be involved. Wnt signaling plays important roles in the differentiation into cardiomyocytes either from tissue regeneration or from pluripotential stem cells,however, its role in differentiation of BMSCs into cardiomyocyte by miR1-2 has not been fully revealed. It is important to determine whether regulation of this pathway after miR1-2 transfection can help to generate sufficient cardiac for therapeutic use in the future.

In this study, we hypothesize that over-expression of miR1-2 in mouse BMSCs can induce their differentiation into cardiomyocytes through Wnt/β-catenin signaling pathway. The effect of miR1-2 in the regulation of the differentiation of BMSCs into cardiomyocytes has been determined and also compared with 5-aza, a positive control that can induce the differentiation of BMSCs into cardiomyocytes.

## Methods

### Animals

Male C57BL/6 mice (4 weeks old) were purchased from the Experimental Animal Center of the Chongqing Medical University (certificate number: SYXK (Chongqing) 2007-0016). All the procedures and experimental protocols were approved by the Experimental Animal Committee of Chongqing Medical University. All the mice were treated in accordance with the guideline for the Care and Use of Laboratory Animals published by the U.S. National Institute of Health.

### BMSCs isolation and culture in vitro

The isolation and culture of BMSCs from C57BL/6 mice was performed as described previously [16]. In brief, after anesthesia, the femurs of mice were quickly taken out, and bone marrow cells were flushed form the bone marrow cavities into beaker with MesenCult basal medium supplemented with Mesenchymal Stem Cell Stimulatory Supplement (Stem cell Technologies, Vancouver, BC, Canada). Bone marrow cells were harvested and plated into the dishes and then incubated with the Dulbecco’s Modified Eagle Medium (DMEM, Hyclone, GE Healthcare Life Sciences,USA) supplemented with 10% fetal bovine serum (Gibco, Burlington, ON, Canada), penicillin (100U/ml)/streptomycin (100μg/ml) (Sigma-Aldrich, St. Louis, MO) in a humidified atmosphere of 5% CO2 at 37 °C. Non-adherent cells were removed carefully after 48h and fresh medium was replaced. When primary cultures became almost confluent, the culture was treated with 0.5ml of 0.25% trypsin containing 0.02% ethylene diamine tetraacetic acid (Sigma-Aldrich, St. Louis, MO) for 1 min at room temperature (25 °C). Cultured BMSCs between passages 3-5 were used for the following experiments.

### BMSCs treatment

The BMSCs were seeded in 60 mm dishes and treated with 5μM 5-aza (Sigma-Aldrich, St. Louis, MO) as previously reported [4] to induce cardiomyocytes differentiation. Then, we transfected 50nM synthetic miR1-2 mimics (mimics-sense (5’-3’):ACAUACUUCUUUAUGUACCCAUA, mimics-antisense(5’-3’):UGGGUACAUAAAGAAGUAUGUUU, Sangon Biotech, Shanghai, China) by Lipofectamine 2000 (Invitrogen, Carlsbad, CA) into BMSCs to over-expression of miR1-2 in BMSCs according to instructions provided by the manufacturer. In the same manner, miR1-2 mimics negative control (miR1-2-mimics NC, NC-sense (5’-3’): UUCUCCGAACGUGUCACGUTT, NC-antisense(5'-3'):ACGUGACACGUUCGGAGAATT, Sangon Biotech, Shanghai, China) was transfected as control. BMSCs treated with DMSO (Invitrogen, Carlsbad, CA) was used as the control also. 1μM LGK-974 (Apexbio, America)was added into BMSCs after miR1-2 mimics transfection at 4 h to inhibit the WNT/β-catenin signaling pathways activities as previously reported [17]. After incubation at 24 h, 48 h and 72 h in a humidified atmosphere of 5% CO2 at 37 °C, the cells were collected for the next experiments.

### Apoptosis assay

The FITC Annexin V Apoptosis Detection kit with PI (Beyotime Biotecnology, Haimen, China) was used to measure the cell apoptosis. After incubation at 48 h and 72 h with 5-aza and miR1-2 mimics, the cells were digested with trypsin and washed twice with PBS and resuspended in Annexin V binding buffer at a concentration of 0.25 ~ 1.0 × 10^7^ cells/ml. The suspension (100 μl) was stained with 5μl of FITC/Annexin V and 10μl of PI, and the cells were gently vortexed and incubated in the dark at 25 °C temperature for 15 minutes. Subsequently, 400μl Annexin V binding buffer was added to each tube and then analyzed by flow cytometry (BD FACSCalibur, USA).

### Real time polymerase chain reaction

To examine the expression of miR1-2, GATA binding protein 4(GATA4), homeobox protein 2.5(Nkx2.5), cardiac troponin I (cTnI), β-catenin, Wnt11, T-cell Factor (TCF), and c-Jun-N-terminal kinase (JNK), we used quantitative real time polymerase chain reaction (qPCR) to measure their mRNA levels. Briefly, total RNAs were isolated and reverse transcribed to cDNA. qPCR was carried out with Tiangen miRNA qPCR kit (FP401, Tiangen, Beijing, China) and Tiangen mRNA qPCR kit (FP302, Tiangen, Beijing, China)with the following parameters: pre-denaturation at 94 °C for 2 min, followed by 40 cycles of denaturation at 94 °C for 10s, annealing at 60 °C for 34s. The primers used in the study are listed in Table 1 and the experiments were performed in triplicate. The levels of miR1-2 were normalized against U6 snRNA. The levels of GATA4, Nkx2.5, cTnI, β-catenin, Wnt11, TCF, and JNK were normalized against GAPDH.

**Table 1:** Primers used in qPCR assays

qPCR primer	
U6	Forward 5’-CCTGCGCAAGGATGAC-3'
	Reverse 5’-GTGCAGGGTCCGAGGT-3'
Nkx2.5	Forward 5’-ACTTCGTGAACTTTGGCGTC -3'
	Reverse 5’-AGGGCATAGTGGGAGCTTTC-3'
cTnI	Forward 5’-TTCTGAGGACTCGTTGCCAG -3'
	Reverse 5’-ATCCACTTTGTCCACCCGAG-3'
GATA4	Forward 5’-TCTCACTATGGGCACAGCAG-3'
	Reverse 5’-GCGATGTCTGAGTGACAGGA-3'
Wnt11	Forward 5’-CCAAATCTCTGCCCTCCTCA-3'
	Reverse 5’-CCTCACCCTTTGACCAACAGA-3'
β-catenin	Forward 5’-GCAGTGAAGAATGCACACGA-3'
	Reverse 5’-CAAGCAAAGTCAGCACCACT-3'
TCF	Forward 5’-ATTAGCGAGAGGGTCTGAGC-3'
	Reverse 5’-AGTTTTGCACACGGTCAGTC-3'
JNK	Forward 5’-TGGAGTCATAAGAGGGCAGC-3'
	Reverse 5’-ACTGCTGTCTGTATCCGAGG-3'
GAPDH	Forward 5’-ACCTGACCTGCCGTCTAGAA-3'
	Reverse 5’-TCCACCACCCTGTTGCTGTA-3'

### Western blotting

Proteins were extracted and quantified as previously reported [18]. Each sample containing equal amounts of protein (30 μg) was subjected to SDS-PAGE and then was electro-blotted onto PVDF membranes. Immunoblotting and detection of the GATA4(1:1000, Abcam, English), Nkx2.5(1:1000,Abcam, English) and cTnI(1:5000) expression were performed. The immunoreactive bands were revealed with enhanced chemiluminescence (Millipore, Billerica, USA), and analyzed using Quantity One Version 4.62 software (Bio-Rad, Richmond, CA). The β-actin was used as the internal controls.

### Data analysis

All statistical analyses were calculated using GraphPad Prism (version6.0, Graphpad Software, SanDiego, USA). Data are expressed as mean ± SEM of three separate experiments. To test the difference between the groups in biochemical measurements for statistical significance, normally distributed data were analyzed by Holm-Sidak tests for multiple group comparisons,and students t-test for two groups comparisons. Data that did not meet the assumptions of analysis were analyzed by the Mann-Whitney U test. We regarded P values of <0.05 as significant.

## Results

### Expression levels of miR1-2 in BMSCs treated with 5-aza and miR1-2

**Fig. 1:**
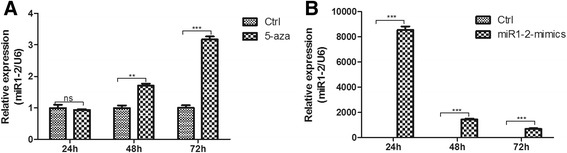
The expression of miR1-2 in BMSCs. The expression of miR1-2 was significantly increased in BMSCs treated with 5-aza and miR1-2 mimics. **a** 5-aza treatment. **b** miR1-2 mimics treatment. ns, *p* > 0.05, **, *p* < 0.01, ***, *p* < 0.001, compared to controls

We used 5-aza to treat BMSCs, we found the expression of miR1-2 did not change at 24 hours, but significantly increased 1.6 times at 48h and 3.0 times at 72h comparing to control after 5-aza intervention, which indicates that the level of miR1-2 was increased in 5-aza-induced BMSCs to differentiate into cardiomyocytes (Fig. 1a). Then, we transfected miR1-2 mimics into BMSCs, 24 hours after the treatment, the expression of miR1-2 in BMSCs was significantly increased, although the level of miR1-2 decreased with time, it was still 600-fold higher than that of the control group after transfection of 72h, (Fig. 1b), indicating that miR1-2 mimics mediated miR1-2 expression in BMSCs.

### Effect of 5-aza and miR-1 mimic on apoptosis in BMSCs

**Fig. 2:**
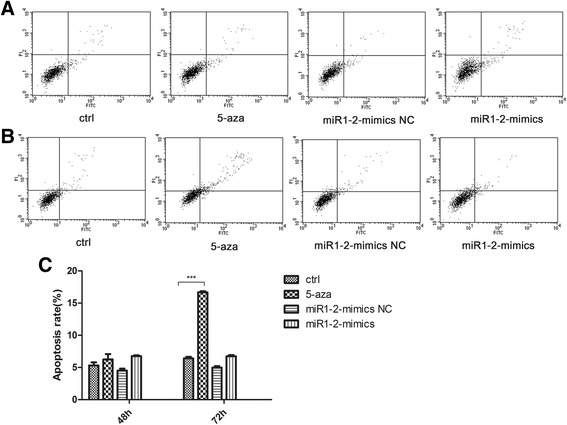
Apoptosis in BMSCs treated with 5-aza and miR1-2 mimics. Flow cytometry assay was used to detect the apoptosis levels. **a** Treatment for 48h. **b** Treatment for 72h. **c** Quantitative analysis (percentage of apoptosis cells versus total cells) showed 5-aza treatment significantly increased the rate of apoptosis while miR1-2 mimics treatment did not increase the rate. ***, *p* < 0.001, compared to controls

We investigated whether 5-aza and miR1-2 mimics treatment could induce apoptosis. Flow cytometry analysis showed that there was no significant difference in the rate of apoptosis in BMSCs after 5-aza and miR1-2 mimics treatment at 48 h (Fig. 2a). However, the rate of apoptosis was significantly increased after 72h treatment with 5-aza (16.51% increase), not in miR1-2mimics group (Fig. 2b), indicating that unlike 5-aza, miR1-2 does not induce cell apoptosis (Fig. 2c)

### Over-expression of miR1-2 induces expression of cardiac-specific genes in BMSCs

**Fig. 3:**
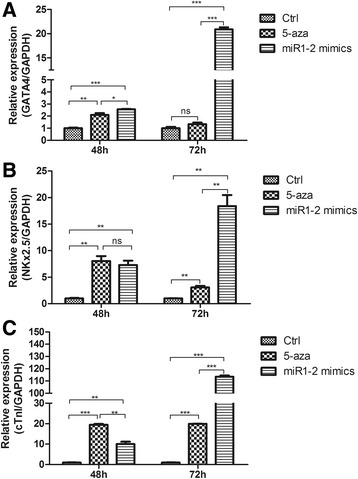
Expressions of cardiomyocyte markers in the BMSCs treated with 5-aza and miR1-2 mimics at 48h and 72h. **a** GATA4 expressions. **b** Nkx2.5 expressions. **c** cTnI expressions. ns, *p* > 0.05, *p < 0.05, **p < 0.01, ***p < 0.001

We then asked whether the over-expression of miR1-2 in BMSCs could induce the differentiation of these cells into cardiomyocytes. The expression levels of several cardiac-specific genes, such as GATA4, cTnI and Nkx2.5 were examined using qPCR. Our data indicated that the expressions of GATA4 (Fig. 3a), Nkx2.5 (Fig. 3b) and cTnI (Fig. 3c) were significantly increased after 48 h treatment with 5-aza, but they decreased with time, the expression of GATA4 even has not significantly increased compared to control group after treatment at 72 h (Fig. 3a). After transfection of miR1-2 mimics at 48 h, the expression of GATA4 (Fig. 3a), Nkx2.5 (Fig. 3b) and cTnI (Fig. 3c) significantly increased also compared to the control, and they increased with time, after 72 h of treatment, the expression of these genes in miR1-2 group were significantly higher than those of the 5-aza group and the control group (Fig. 3). These results indicated that over-expression of miR1-2 could induce BMSCs differentiation into cardiac cells by enhancing the expression levels of the cardiac genes.

### Over-expression of miR1-2 activates Wnt/β-catenin signaling pathway

**Fig. 4:**
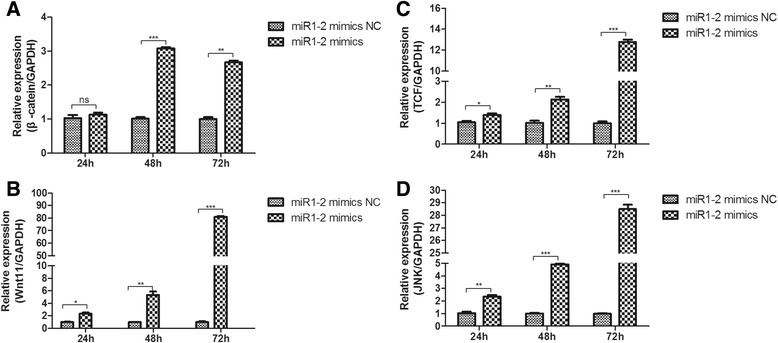
Expression levels of β-catenin, Wnt11, TCF, and JNK in BMSCs. The expression levels of β-catenin (**a**) Wnt11 (**b**) TCF (**b**) and JNK (**d**) were significantly increased in the BMSCs after miR1-2 mimics treatment. ns, *p* > 0.05, *, *p* < 0.05, **, *p* < 0.01, ***, *p* < 0.001, compared to miR1-2 mimics NC

Previous studies strongly implied that miR-1 promoted cardiac differentiation in human embryonic stem cells, cardiac stem cells, or cardiac progenitor cells via regulating the activity of the Wnt/β-catenin signaling pathway. We therefore asked whether the over-expression of miR1-2 induces cardiac differentiation of mouse BMSCs through the Wnt/β-catenin signaling pathway. For this purpose, we measured the transcription levels of upstream protein including β-catenin, wnt11, TCF, and JNK of Wnt/β-catenin pathway in BMSCs after miR1-2 mimic treatment. The results showed that the relative expression of Wnt11 (Fig. 4b), TCF (Fig. 4c), and JNK (Fig. 4d) was significantly increased after 24 h treatment with miR1-2 mimics compared to the miR1-2 mimics NC. Though the expression of β-catenin was not increased at 24 h, but it significantly increased at 48 h and 72h (Fig. 4a). These results indicated that Wnt signaling pathway may play important roles in the differentiation of BMSCs into cardiomyocytes by miR1-2 induced.

### miR1-2 induces cardiac differentiation through Wnt/β-catenin signaling activation

**Fig. 5:**
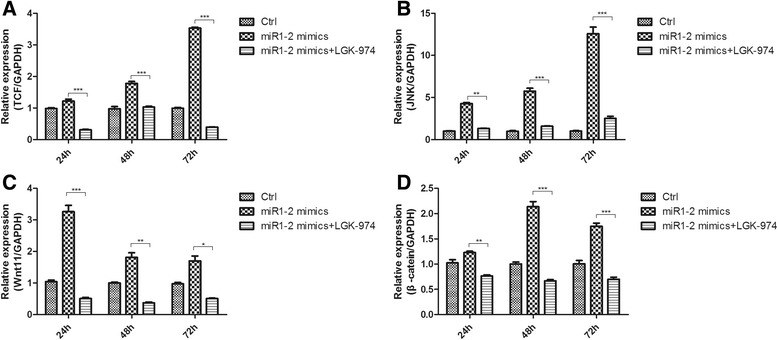
Wnt/β-catenin signaling pathways were activated by miR1-2mimics treatment but blocked by LGK-974 in BMSCs. **a** TCF expressions. **b** JNK expressions. **c** Wnt11 expressions. **d** β-catenin expressions. *, *p* < 0.05, **, *p* < 0.01, ***, *p* < 0.001, compared to miR1-2 mimics

**Fig. 6:**
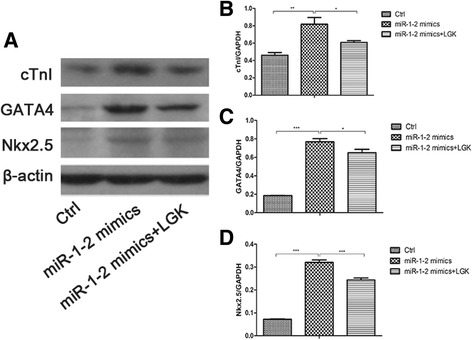
Expression of cardiac protein in BMSCs after miR1-2mimics treatment. **a** Western blot band. **b**, **c**, **d** WB analysis confirmed that miR1-2mimics treatment increased the expression of cTnI(B), GATA4(C), and Nkx2.5(D) in mouse BMSCs, **, *p* < 0.01, ***, *p* < 0.001, compared to controls. While using LGK-974(Wnt inhibitor) exhibited a distinct reduction of the protein expression of cTnI(B), GATA4(C) and Nkx 2.5(D). *, *p* < 0.05, ***, *p* < 0.001, compared to miR1-2 mimics

We then asked whether the increased level of cardiac-specific genes induced by the over-expression of miR1-2 is through the Wnt signaling. For this purpose, we used LGK-974, an inhibitor of Wnt/β-catenin signaling pathway to suppress the activation of Wnt/β-catenin signaling pathway. As a result, the relative expression of β-catenin, JNK, Wnt11 and TCF was significantly increased after miR1-2 mimic treatment, but the levels of β-catenin together with JNK, Wnt11 and TCF were significantly decreased after adding LGK-974 (Fig. 5). In parallel to the data from qPCR analysis, WB analysis also confirmed that after treated with miR1-2mimics at 72h, BMSCs increased the expression of the protein of GATA4, NKx 2.5 and cTnI, while treated by Wnt inhibitor, the BMSCs exhibited a distinct reduction of the protein of GATA4, Nkx2.5 and cTnI, compared to the miR1-2 mimics group without Wnt inhibitor treatment (Fig. 6)

## Discussions

It has been documented that BMSCs were capable of turning differentiating into cardiomyocytes, endothelial cells, and vascular smooth muscle cells [2–4, 19]. In this study, we found that BMSCs treated with miR1-2 mimics displayed the increased expression of cardiac-specific markers such as GATA4 and Nkx2.5. The levels of those cardiac-specific markers were significantly higher than those in 5-aza group. This was consistent with the previous reports that miR-1 induced the expression of several cardiomyocyte markers, including Nkx2.5, GATA4, cTnI, and Cx43 in MSCs^14^. It was reported that translocation of Nkx2.5 and GATA4 to the nucleus can drive BMSCs to Cardiac phenotype [19], miR-1 could upregulate Nkx2.5 to promote Cardiac differentiation [20]. We also observed that the apoptosis rate of the miR1-2 mimics group was significantly lower than the 5-aza group. Carley et al also reported that miRNA1 transfected embryonic stem cells could inhibit apoptosis by modulating the PTEN/Akt pathway in the infarcted heart [21].All of these results suggested that miR1-2 could induce the differentiation of BMSCs into cardiomyocytes, and this method may be more effective and less cytotoxic.

Wnt signaling pathway plays an essential role during embryonic and postnatal cardiomyocytes development. Previous studies has showed that β-catenin in Drosophila could promote the occurrence of the heart [22].Wnt11 could regulate cell adhesion and affect the formation of cardiac linear tube, knockdown wnt11 would lead the defects of ventricular outflow tract, and the reduction of cardiac trabecular in mouse [23]. The Wnt signaling pathway also simultaneously regulates the proliferation and the differentiation of BMSCs [24]. Moreover, miRNA could regulate gene expression and act as important factors in the cardiomyocytes development and the differentiation of stem cell [12–14].Therefore, these observations raised an interesting question whether miRNA could modulate signaling pathway to regulate the differentiation of stem cell. Recently, accumulated evidences indicated that miRNA could regulate various bioprocesses by targeting Wnt signaling pathways. For example, miR218 was reported to be able to promote the differentiation of BMSCs by activating a positive Wnt signaling loop [25]. miR27 inhibits the gene expression of adenomatous polyposis coli leading β-catenin accumulation and thus Wnt signaling pathways activation to promote osteoblast differentiation[26]. Tcf-1 is a critical target gene of the Wnt/β-catenin signaling pathways [27]. Inhibiting low density lipoprotein receptor-related protein 6 by miR30e over expression significantly downregulates β-catenin/Tcf transcriptional activity and dramatically inhibits osteoblasts differentiation [28]. All these results are accordant with our findings in this study that the over-expression of miR1-2 activated Wnt/β-catenin pathway as evidenced by the findings that the expression of β-catenin, Wnt 11, TCF, and JNK were significantly increased. After using LGK-974, a highly potent and selective Wnt signaling antagonist [29] to block the Wnt/β-catenin signaling, the activity of the Wnt/β-catenin signaling pathways and the expression of cardiac-genes both decreased. These data indicated miR1-2 might promote the differentiation of BMSCs into cardiomyocytes via activation of the Wnt/β-catenin signaling pathways. However, we did not find a decrease in expression levels in the molecules of the detected Wnt signaling pathways. It’s controversy to the miRNA function post-transcriptionally by interacting directly with 3′-UTRs of mRNAs to repress their expression by translational inhibition, mRNA degradation, or both [30].Maybe, it suggests the existence of undefined miR1-2 target genes, which could inhibit Wnt signaling. It has been reported that miR-335-5p activated Wnt signaling and promoted osteogenic differentiation by downregulating Dickkopf-related protein 1 (DKK1), an inhibitor of Wnt signaling [31]. Yalan Yang et al also found that miR-1/206 targeted Secreted frizzled-related protein one (SFRP1), another inhibitor of Wnt signaling to promote skeletal muscle development [32].

Although in our study, BMSCs treated with miR1-2 expressed cardiac-specific genes, these cells still lacked the morphology of cardiomyocytes and did not beat by themselves, which were observed in studies with embryonic stem cells [33]. In addition, the undefined target genes of microRNA1-2 warrant further investigation.

## Conclusions

In summary, our study demonstrated that the over-expression of miR1-2 in BMSCS induced them toward cardiomyocyte differentiation through activating Wnt/β-catenin signaling pathways, and miR1-2 can induce BMSCs differentiation into cardiomyocytes more effectively with a less cytotoxicity than that of 5-aza. These findings will greatly improve our understanding of the roles of miRNA in cardiomyocyte differentiation of stem cells and improve the effects of BMSCs-based therapy for damaged myocardial repair and regeneration.

## Abbreviations

microRNA: micro ribonucleic acid
BMSCs: Bone marrow-derived mesenchymal stem cells
cTnI: Cardiac troponin I
Nkx2.5: Homeobox protein 2.5
GATA4: GATA binding protein 4
JNK: c-Jun-N-terminal kinase
TCF: T-cell Factor
